# Universal coverage and economic burden from epidemiological changes of diabetes in Latin America

**DOI:** 10.7189/jogh.06.020309

**Published:** 2016-12

**Authors:** Armando Arredondo

**Affiliations:** Instituto Nacional de Salud Pública, Cuernavaca Mor, México

Despite the increased coverage schemes of universal health insurance, unresolved challenges still remain in the current health care model. The expected epidemiological changes for chronic diseases in Latin American countries (LACs), will lead, in economic terms, to catastrophic expenditures for the health systems (more than 10% of the health budget) and for patients (more than 30% of the household income). Moreover, the governments, institutions and societies of these countries will have to face strong competition in the allocation of resources to universal coverage for other diseases [1].

Undoubtedly, talking about chronic diseases, diabetes is a global public health problem of great relevance in LACs. In this sense, we will take diabetes as a tracer problem of chronic disease challenges for universal coverage schemes in these countries. The rapid growth of diabetes is a global event with broad challenges for public health systems at a world level. Diabetes and its complications are a great economic challenge for any scheme of universal coverage, particularly when it is present in older people [2]. The challenges increase because in LACs, financial resources for health services are more and more limited, a great part of these is allocated to curing and few resources are used for prevention; on the other hand, a culture of self–care and healthy behaviors is not very present in these countries [3].

We want to highlight the fact that in LACs, just as in other countries, we are also facing a global problem that is generating a high catastrophic expenditure for all of those who are involved. For example, in the case of Mexico, the same methodology of a study conducted in 2004 was used to identify the costs generated in 2015 by diabetes in the national population [4]. Results show the high impact on public health systems, but also on patients’ pockets. Indeed, the demand for health care for older adults goes beyond the capacity of the public health system and patients end up financing most of the care for diabetes and its complications [5]. Indeed, of every US$ 100 spent on diabetes in Mexico in 2015, patients contributed US$ 54 and the public health system contributed US$ 46. This evidence has considerable implications in terms of equity and access to public health programs. In this sense, patients’ catastrophic expenditures will increase and, above all, the high costs of lost productivity attributable to temporary disability, permanent disability and premature death, generated by diabetes [6].

The main objective of this essay is to highlight that the epidemiological and economic trends reported by several studies in different years (2000, 2010 and 2025), show a constant increase, despite the efforts made by the new universal coverage schemes to reduce the impact of diseases such as diabetes. Also, we highlight the achievements and challenges of universal coverage and how it relates to the prevention of diabetes, and finally, we conclude with a list of possible strategies for the solution of this problem in LACs.

## WHAT IS THE PROBLEM?

In the context of universal coverage schemes without substantive changes in the health care model of, diabetes has assumed one of the top trends in morbidity and mortality in most countries of the world, generating great challenges for medicine and public health. The World Atlas of Diabetes registry for 2015, reports 415 million adults with diabetes [7]. This number will continue to Increase globally due to an aging population, growth of population size, urbanization and high prevalence of obesity and a sedentary lifestyle.

With respect to the place of diabetes in the epidemiological burden to the Latin American region, a recent study reported diabetes and other chronic diseases as main causes of mortality for all LACs. The relative weight of these diseases on the total burden are in a minimum range of 62% in Costa Rica, with a maximum range of 84% in Chile [8]. With respect to the impact on DALYS for 2011, the main reported causes are major unipolar depression, alcohol consumption, asthma, dental cavities, cardiovascular diseases and diabetes. For example, in the results by country, Brazil had a greater impact, with a total of 37.5 million DALYS, a rate of 232 per one thousand inhabitants.

In Brazil, as in most LACs, diabetes mellitus was in first place, having 5.1% of DALYS, followed by ischemic heart disease (5%), cerebrovascular diseases (4.6%) and depressive disorders and asphyxia at birth (3.8%). In women, diabetes mellitus was in first place (6.9%), depressive disorders in second (6.3%) and cerebrovascular diseases in third place (4.5%). In men, assaults stood out (5.6%), ischemic heart disease (5.6%), cerebrovascular diseases (4.6%) and diabetes (4.4%) [8].

With respect to the epidemiological burden at the global level in 2015, the top ten countries, from greater to lesser impact, were (millions of adults with diabetes): China (109.6), India (69.3), USA (29.3), Brazil (14.3), Russia (12.1), Mexico (11.5), Indonesia (10.0), Egypt (7.8), Japan (7.2), and Bangladesh (7.1) Evidently, the DALYS with greater impact are also for these 10 countries, led by China and India at the global level, and by Brazil and Mexico at the level of LACs [9].

Moreover, from the perspective of the epidemiological transition, the latest Global Burden of Disease study (GBD) reported that by 2010, diabetes [10], as a tracer of the epidemiological transition in the world, is one of the biggest challenges being faced by health systems and society. The challenges get more complicated, not only in terms of mortality but also by generating growth and diversification in the demand for health care services for resolution, in the framework of the health transition.

**Figure 1:**
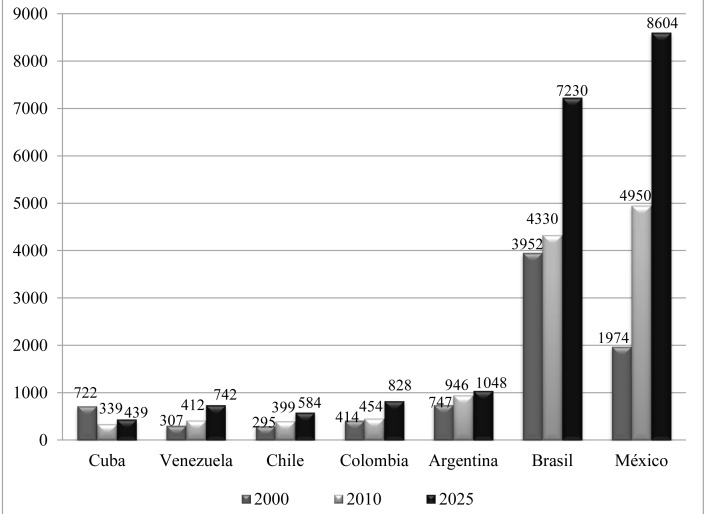
Comparative data of economic burden from epidemiological trends (observed 2010, 2010, and expected 2025) in diabetes for selected Latin American countries (millions of US$). Source: Developed by author with data from references [4,9–11].

With regards to diabetes, contrary to the main purpose of the strategy of universal coverage, the epidemiological transition phenomenon in economic terms, represents a heavy burden in direct costs to the users’ pockets, to the health system and society, and indirect costs attributable to premature mortality, temporary disability and permanent disability attributable to the complications of diabetes [11]. Indeed, integrating a database of several published studies, we analyze findings in seven Latin American countries selected under criteria of diabetes prevalence, data on the epidemiological and economic burden of diabetes, and income level: Cuba, Venezuela, Chile, Colombia, Argentina, Brazil and Mexico. The comparative analysis of the 7 countries includes epidemiological and economic trends reported by other studies for the years 2000 and 2010 and expected for 2025 [12]. The costs from epidemiological changes observed in a group of countries selected for this essay, have increasing trends if current epidemiological conditions and current models of care are maintained, mainly in Mexico, Argentina and Brazil (**Figure 1**).

On the other hand, in Latin America and the Caribbean, even with new universal coverage schemes, many people with diabetes have limited access to health care; this means that indirect costs may exceed direct health care costs. In terms of the response of the health system, in recent years, health systems in most LACs have undertaken adjustment, changes or reforms in national health programs trying to meet the goal of universal health insurance. Longer life expectancy and fewer families who are impoverished due to health reasons, are some of the results obtained in recent years following the adoption of universal health insurance in these countries. Indeed, since 2005, the new Health Insurance Program in Argentina has helped introduce historical changes in universal coverage by the health system [13]. In the case of Brazil, the tax–funded Unified Health System modernized the Brazilian health system, creating a national coordinated service that the entire population can access. Investing heavily in a “Family Health” primary care strategy in particular has been the vehicle used to carry out major reforms allowing families greater access to health care through home visits and community activities for better health.

In Chile, the “Social Health Insurance” program ensures nearly universal health coverage for its 17 million inhabitants. From 2005, all Chileans have access to a basic package that guarantees treatment for 80 health problems [14], establishing maximum waiting times for treatment and discretionary spending. In Colombia in 1991, after establishing the right to health in its constitution, 20 years later, access to health services has improved considerably thanks to a national system of subsidized health insurance [15].

In the case of México, covering more than 50 million people, the Popular Health Insurance, with universal coverage strategies, promotes access to health care for all those who lack social security. At the heart of the 2003–2018 health reform, this coverage package includes more than 230 primary and secondary treatments for the entire population, including interventions for diabetes and its major complications [16].

Despite advances in coverage under schemes of “Universal Health Insurance” in all those countries, the epidemiological and economic burdens of problems such as diabetes, far from resolved, continue with constant incremental trends which can be seen in **Figure 1**. Expectations are nothing favorable if major changes are not implemented in the models of care. The problem is that even with more coverage and access to health care, the health care model remains the same as when it began in the 1940s. This model is based on a fragmented scheme with several institutions providing health care for people in the formal economy (social security institutes) vs institutions for the population in the informal economy (ministries of health) [17]. These institutions, dated from the 1940s, provide health services based on a model of care with a biomedical curative approach. In this sense, most of the national health expenditure goes to curative health programs (90–95% depending on the country) and the results in terms of benefits for chronic diseases such as diabetes, have not been favorable.

Summarizing, we note that aside from interventions from universal coverage programs aimed at diabetes, it is necessary to review and adjust prevention strategies. We already have enough evidence on prevention strategies, costs and effectiveness in all regions of the world. In the case of LACs, this review has defined a list of 10 major prevention strategies (**Table 1**). These strategies consider, from different perspectives, the effects of changes in lifestyle and/or the use of metformin or other drugs used to control blood glucose levels, as the best options.

**Table 1:** Diabetes prevention strategies and implementation – challenges in Latin American countries

Prevention strategy from universal coverage	Implementation challenges
Institutional intervention for lifestyle changes and/or use of effective pharmacological agents to prevent damage–complications in patients with diabetes or to delay the appearance of the disease in pre–diabetic patients.	Difficulties and inadequacy of international standards in defining lifestyle indicators from a biomedical approach. This leads to high rates of treatment desertion due to problems related to institutional cultural aspects, drug availability and resistance to changes in lifestyle. Pre–diabetes programs are only mentioned but not implemented in practice, mainly due to lack of resources.
Intra–institutional and inter–sectorial programs promoting changes in lifestyle through mass media programs.	Intra–institutional and cultural barriers in the definition, promotion and communication of lifestyle indicators by country or region. Because of the fragmentation of the health system, each institution implements its program according to its resources and organizational culture. When involving inter–sectorial actions that require participation of the health and education sectors there is no agreement or coordination.
Community programs for lifestyle changes centered on eating habits and diet. Directed to 4 age groups: children, adolescents, young adults and older adults	Lack of knowledge and / or limited availability of healthy foods. Conflict between suggested diets and consumption patterns and social and cultural determinants that are difficult to change.
Community programs for changes in lifestyle focusing on physical activity	Lack of time and space for physical activity. Obesogenic environments determined by cultural aspects depending on the country or region.
Programs to eliminate obesogenic environments at macro, meso and micro levels.	No proposal for intervention vs obesogenic environments involving actors from the health–education and environment areas, working together.
Development of an integrated multicenter, multidisciplinary and inter–sectorial approach for prevention of diabetes and its complications.	A biomedical approach continues to dominate, which is fragmented within each institution and without involvement of social science disciplines. In health teams, doctors and other professionals from the health sciences predominate but only rarely involve psychologists, sociologists or anthropologists, despite the large indigenous population that generally does not speak Spanish and with habits and customs that health personnel do not know.
Community prevention programs as part of universal coverage.	Lack of efficiency in the allocation of resources to start a phase of universal coverage strategies. Problems of financial sustainability for consolidation stages of programs focused on diabetes prevention.
National strategies for prevention of diabetes and obesity involving all actors.	Absence or very low participation of key stakeholders of civil society, community leaders and entrepreneurs.
Strategy to impact on the assessment of prevention interventions.	Lack of financial resources, research teams and a culture of accountability at the institutional or national/international levels.
Partnership Program for the Health System and Companies/Institutions working on prevention of complications and to reduce disability from diabetes.	The health system has been unable to build solid partnerships with companies to develop these programs. The social costs of disability attributable to complications continue to grow in all countries.

The challenge for the universal coverage strategy in LACs is the design and implementation of effective prevention strategies. **Table 1** also highlights the main challenges or problems that must be solved for a more effective prevention of diabetes. These challenges are those subsequently taken up for analysis of possible alternative solutions.

## SUGGESTIONS FOR A POSSIBLE SOLUTION

Increased coverage by “Universal Health Insurance” schemes has not been sufficient to meet the challenges of chronic health problems in LACs. In terms of changes in the health system with any scheme of universal coverage, the main adjustment should be related to the transition from one system of care based on a biomedical, curative, fragmented and inequitable model toward a socio–medical model, preventive medicine, which is comprehensive and equitable. This will enable more effective detection and control with a consequent decrease in the effect of complications and treatment desertion. In most LACs, of every 100 patients with diabetes, only 50 are diagnosed and of these 50, only 30 remain in control. With a more effective universal coverage, these indicators should change with new strategies for detection and control.

Effective universal coverage involves approaching diabetes from an interdisciplinary perspective to promote a change in the concept and determinants of diabetes, as well as a change in the social meaning of the disease and greater involvement of users, civil society and businesses. It requires allocating more resources to design, implement and monitor strategies to move from addressing diagnosed patients to strategies for the pre–diabetes population. In all LACs, there is little or no intervention for this population.

Development and validation of new methods are need to evaluate the epidemiological and economic burdens in terms of direct costs of care and indirect costs (temporary disability, permanent disability and premature mortality). For more effective coverage schemes, in all LACs it is necessary to adjust/implement new models of care and health management that can respond to the diversification and quantity of health services that will be generated by the epidemiological transition in chronic diseases, particularly in patients with diabetes or hypertension.

## WHAT NEEDS TO HAPPEN NEXT?

As part of an effective universal coverage scheme by universal health insurance, the proposed changes in reforms or adjustments in the health system in LACs, should put emphasis on changes in the health care model with a greater focus on the level of primary prevention. The following strategies are highlighted in order of priority:

- The current model of care must go through a detailed review, to propose changes in the physical infrastructure and the training of health personnel with a focus on prevention. We must develop infrastructure to expand screening programs, for more detection, prevention and control. We also have to implement changes in the continuing education programs for health personnel to enable a greater focus on primary care (mainly for physicians and nurses). We also suggest integrating social science professionals into health programs (medical anthropologists and medical sociologists) to form part of the health team to implement new strategies for detection, prevention and control of chronic diseases.
- Develop new financing schemes with greater allocation of resources to new programs for screening and prevention in the pre–diabetes population.
- Design and implement systems for epidemiological surveillance and monitoring of the economic burden for a periodic measurement that allows us to know and assess (preferably on an annual or biannual basis) the impact of new strategies on epidemiological trends as indicators of direct and indirect costs.
- Establish patterns of resource allocation to ensure the financial requirements to address diabetes based on expected demand. These patterns must integrate indicators on clinical efficiency (inpatient and outpatient cases), epidemiological efficiency (new cases of diabetes from expected trends in the short term), organizational efficiency (number of cases to be taken care of by level of care) and economic efficiency (average cost of case management by level of care).
- Knowledge of the relative weight of the management of diabetes based on the annual family income, as well as required knowledge of the cost of complications to the users, should be made available through a bulletin sent to patients and their relatives, and to the community as a whole.

**Figure:**
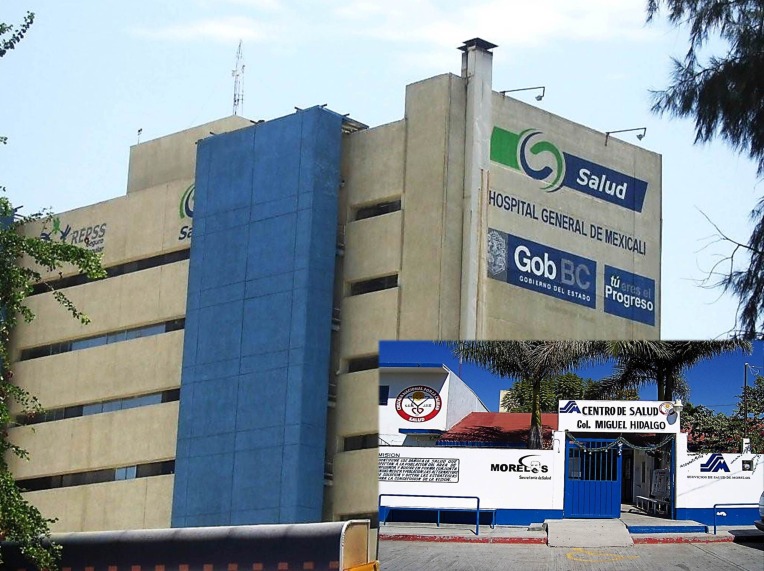
Photo: The difference infrastructure for curative care vs. primary care in Mexico. Courtesy of the author.
- A list of recommendations is needed to promote greater self–care, monitoring of risk factors and the benefits of carrying out these measures, and more importantly to avoid falling into a catastrophic situation because of the costs of diabetes (to avoid an impact of >30% of the family income).
- As a “Regional Observatory Citizen of Diabetes”, social civil organizations could suggest and develop follow–up programmes for the costs of diabetes in different public and private health institutions. The Observatory should function as a checking system that would monitor how much is being spent on managing diabetes and what the money is being spent on.
- With regards to the indirect costs of premature mortality and temporary and permanent disability attributable to diabetes, companies must establish new partnerships and agreements with the health system and workers to have positive gain in economic competitiveness and labor productivity. This will require developing new programs in the workplace for increased detection, prevention, treatment and control of diabetes and its complications.
- In most LACs, the strategy to expand coverage through various schemes of “Universal Health Insurance” presents evidence of benefit and greater access to health care in general but with some limitations in the current shape of the health systems. Indeed, the structure of the health system in which they operate such strategy is the very structure of the past half century, with a focus on curative care.
- The groups of patients with diabetes could collaborate on joint actions with the health system in order to promote universal coverage schemes, new actions based on the perspective of “health behavior” with a vision of diabetes as a “life condition”, more than a health problem.
- All these strategies should place a greater emphasis on actions to move from a model of biomedical care based on curative medicine to one of universal insurance focused on socio–medical health care based on preventive medicine. Like this, LACs can more effectively face the current public health challenges for chronic diseases like diabetes.
